# Brain dysfunction in chronic pain patients assessed by resting-state electroencephalography

**DOI:** 10.1097/j.pain.0000000000001666

**Published:** 2019-07-22

**Authors:** Son Ta Dinh, Moritz M. Nickel, Laura Tiemann, Elisabeth S. May, Henrik Heitmann, Vanessa D. Hohn, Günther Edenharter, Daniel Utpadel-Fischler, Thomas R. Tölle, Paul Sauseng, Joachim Gross, Markus Ploner

**Affiliations:** a Department of Neurology, School of Medicine, Technical University of Munich, Munich, Germany; b TUM-Neuroimaging Center, School of Medicine, Technical University of Munich, Munich, Germany; c Department of Anesthesiology, School of Medicine, Technical University of Munich, Munich, Germany; d Department of Psychology, Ludwig-Maximilians-Universität München, Munich, Germany; e Institute for Biomagnetism and Biosignalanalysis, University of Münster, Münster, Germany; f Centre for Cognitive Neuroimaging, University of Glasgow, Glasgow, United Kingdom

**Keywords:** Chronic pain, Electroencephalography, Oscillations, Synchrony, Brain networks, Theta, Gamma

## Abstract

Chronic pain is a common and severely disabling disease whose treatment is often unsatisfactory. Insights into the brain mechanisms of chronic pain promise to advance the understanding of the underlying pathophysiology and might help to develop disease markers and novel treatments. Here, we systematically exploited the potential of electroencephalography to determine abnormalities of brain function during the resting state in chronic pain. To this end, we performed state-of-the-art analyses of oscillatory brain activity, brain connectivity, and brain networks in 101 patients of either sex suffering from chronic pain. The results show that global and local measures of brain activity did not differ between chronic pain patients and a healthy control group. However, we observed significantly increased connectivity at theta (4-8 Hz) and gamma (>60 Hz) frequencies in frontal brain areas as well as global network reorganization at gamma frequencies in chronic pain patients. Furthermore, a machine learning algorithm could differentiate between patients and healthy controls with an above-chance accuracy of 57%, mostly based on frontal connectivity. These results suggest that increased theta and gamma synchrony in frontal brain areas are involved in the pathophysiology of chronic pain. Although substantial challenges concerning the reproducibility of the findings and the accuracy, specificity, and validity of potential electroencephalography-based disease markers remain to be overcome, our study indicates that abnormal frontal synchrony at theta and gamma frequencies might be promising targets for noninvasive brain stimulation and/or neurofeedback approaches.

## Abstract 2

Resting-state electroencephalography reveals increased synchrony at theta and gamma frequencies in frontal brain areas and global network reorganization at gamma frequencies in chronic pain patients.

## 1. Introduction

Chronic pain is a disease characterized by ongoing pain and significant sensory, cognitive, and affective abnormalities^49,77^ that have detrimental effects on quality of life. Affecting between 20% and 30% of the adult population,^8,35^ chronic pain is a leading cause of disability worldwide.^27^ Thus, advances in the understanding and treatment of chronic pain are urgently needed.

Studies in animals and humans have revealed that chronic pain is associated with significant structural and functional changes of the brain.^2,38,55^ In particular, the prefrontal cortex and subcortical brain areas including amygdala, hippocampus, and striatal areas have been implicated in chronic pain.^2,3,31,47,55,62,72^ Further insights into the brain mechanisms of chronic pain not only promise to advance the understanding of the underlying pathophysiology but could also be clinically highly useful. In particular, a brain-based marker of chronic pain could be immensely helpful for the diagnosis, prognosis, classification, prevention, and treatment of pain.^15,71^ Accordingly, the feasibility, limitations, and perspectives of brain-based markers of pain are currently intensively discussed in the pain research community^15,50,64^ and beyond.^43,56,84^ Recent functional magnetic resonance imaging (fMRI) studies have made important first steps towards such a brain-based marker of experimental^80^ and chronic pain.^44,45^

Using electroencephalography (EEG) to assess abnormalities of brain function and to establish a brain-based marker of chronic pain is particularly appealing because it is safe, cost-effective, broadly available, and potentially mobile. Moreover, an EEG-based marker of chronic pain might not only be helpful for the diagnosis and classification of chronic pain but might itself represent a target for novel therapeutic strategies such as neurofeedback^63^ or noninvasive brain stimulation techniques.^54^ As a potential first step in that direction, some EEG studies have shown a slowing of neural oscillations together with an increase of oscillatory brain activity at theta frequencies (4-8 Hz) in chronic pain patients.^58,76^ These observations have been embedded in the thalamocortical dysrhythmia (TCD) model of neuropsychiatric disorders.^41^ In this model, abnormal thalamocortical theta activity yields abnormal oscillations at gamma (>30 Hz) frequencies, which eventually result in different neuropsychiatric symptoms including ongoing pain. This model is highly appealing, but evidence is sparse, contradictory, and mostly confined to small groups of patients suffering from neuropathic subtypes of chronic pain.^52,53^ Thus, a general EEG-based marker of chronic pain remains to be demonstrated.

In this study, we aimed to systematically and extensively exploit the potential of EEG to determine abnormalities of resting-state brain activity as a potential brain-based marker of chronic pain. In a large cohort of chronic pain patients and age- and sex-matched healthy controls, we analyzed global and local measures of oscillatory brain activity. Moreover, we performed connectivity analyses using phase-based and amplitude-based measures in source space as well as graph theory–based network analyses. In a univariate approach, we statistically compared these measures between patients and healthy controls. Moreover, in a multivariate machine learning approach, we tested whether patterns of these measures allow to distinguish between chronic pain patients and healthy controls.

## 2. Materials and methods

### 2.1. Participants

One hundred one patients (age 58.2 ± 13.5 years [mean ± SD], 69 female) suffering from chronic pain and 84 age- and sex-matched healthy control participants (age 57.8 ± 14.6 years, 55 female) participated in the study. Inclusion criteria for patients were a clinical diagnosis of chronic pain, with pain lasting at least 6 months and a minimum reported average pain intensity ≥4/10 during the past 4 weeks (0 = no pain, 10 = worst imaginable pain). Exclusion criteria were acute changes of the pain condition during the past 3 months, for example, due to recent injuries or surgeries. Further exclusion criteria were major neurological diseases such as stroke, epilepsy, or dementia, major psychiatric diseases aside from depression, and severe general diseases. Finally, patients on medication with benzodiazepines were excluded, other medication was not restricted, and patients' medication was maintained. Demographic and clinical details of participants are shown in Tables 1 and 2, respectively. In summary, we included 47 patients with chronic back pain, 30 patients with chronic widespread pain, 6 patients with joint pain, 5 patients with unspecific neuropathic pain, 7 patients with postherpetic neuralgia, and 6 patients with polyneuropathic pain. All participants provided written informed consent. The study was approved by the ethics committee of the Medical Faculty of the Technical University of Munich and conducted in accordance with the relevant guidelines and regulations. A power analysis for independent 2-sample *t*-tests using G*Power^22^ showed that the number of participants allowed for detecting differences between groups of at least medium effect size (Cohen's *d* = 0.5) with a statistical power of 0.9.

**Table 1:**
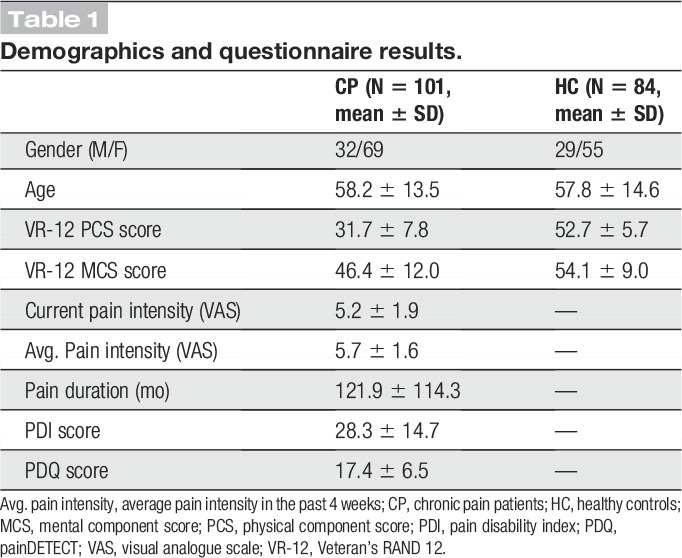
Demographics and questionnaire results.

**Table 2:**
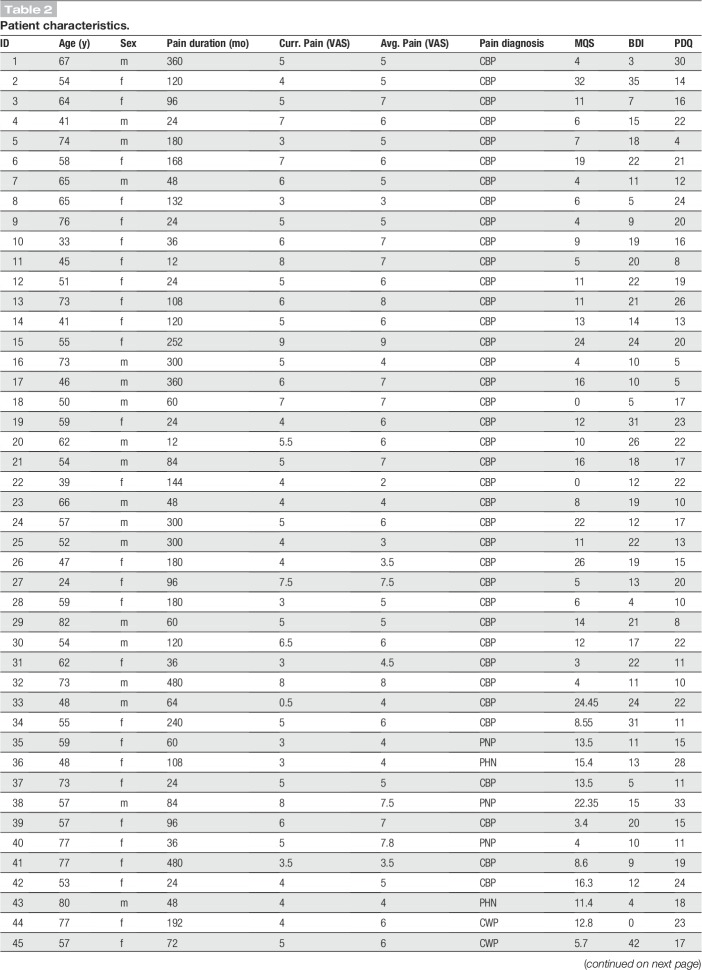

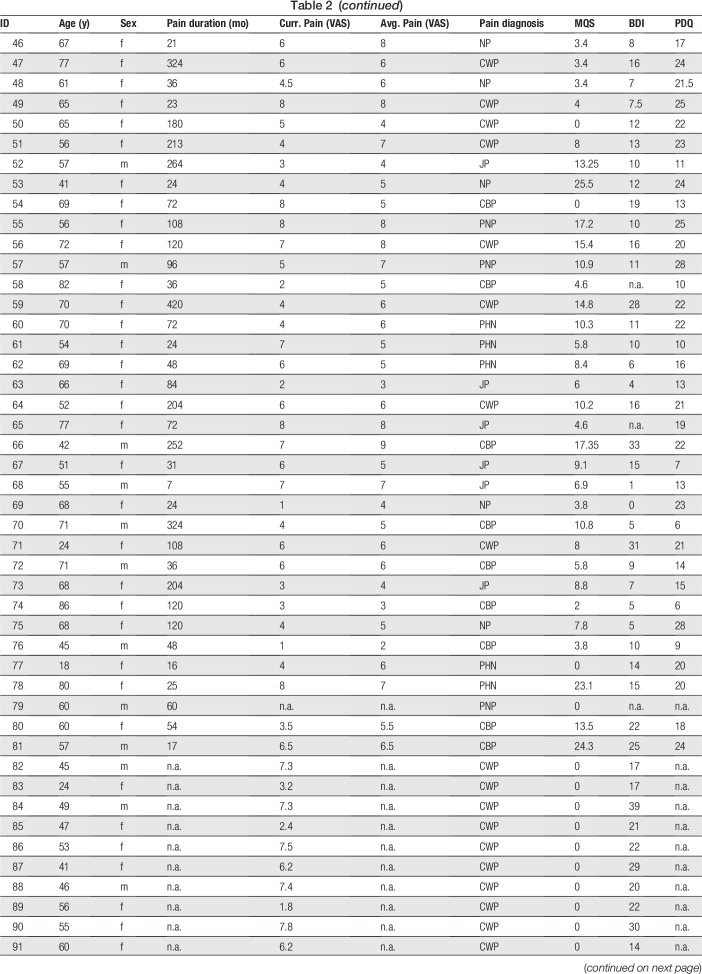

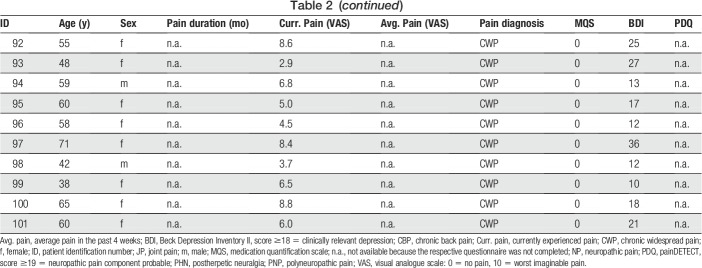
Patient characteristics.

### 2.2. Recordings

Brain activity was recorded using EEG. Recordings were performed during the resting state, ie, participants were asked to stay in a relaxed and wakeful state, without any particular task. Electroencephalography data were recorded with eyes closed and eyes open for 5 minutes each. Because the eyes-closed condition showed better data quality and less muscle artifacts, analyses were focused on this condition.

Electroencephalography data were recorded using 64 electrodes consisting of all 10-20 system electrodes and the additional electrodes Fpz, CPz, POz, Oz, Iz, AF3/4, F5/6, FC1/2/3/4/5/6, FT7/8/9/10, C1/2/5/6, CP1/2/3/4/5/6, TP7/8/9/10, P5/6, and PO1/2/9/10 plus 2 electrodes below the outer canthus of each eye (Easycap, Herrsching, Germany) and BrainAmp MR plus amplifiers (Brain Products, Munich, Germany). All EEG electrodes were referenced to FCz and grounded at AFz. Simultaneously, muscle activity was recorded with 2 bipolar surface electromyography (EMG) electrode montages placed on the right masseter and neck (semispinalis capitis and splenius capitis) muscles^14^ and a BrainAmp ExG MR amplifier (Brain Products). The EMG ground electrode was placed at vertebra C2. All data were sampled at 1000 Hz (0.1 μV resolution) and band-pass filtered between 0.016 and 250 Hz. Impedances were kept below 20 kΩ.

Before the EEG recordings, patients completed the following questionnaires to assess pain characteristics and comorbidities: short-form McGill pain Questionnaire,^48^ Pain Disability Index,^18^ painDETECT (PDQ),^24^ Beck Depression Inventory II (BDI-II),^6^ State-Trait-Anxiety Inventory,^65^ and the Veteran's RAND 12-Item Health Survey (VR-12).^61^

### 2.3. Preprocessing

Preprocessing was performed using the BrainVision Analyzer software (Brain Products). Data were downsampled to 250 Hz. For artifact identification, a high-pass filter of 1 Hz and a 50-Hz notch filter for line noise removal were applied to the EEG data. Independent component analysis was performed,^34^ and components representing eye movements and muscle artifacts were identified based on time courses and topographies. Furthermore, time intervals of 400 ms around data points with signal jumps exceeding ±100 µV were marked for rejection. Finally, all data were visually inspected and remaining bad segments marked. Subsequently, independent components representing artifacts were subtracted from the raw, unfiltered EEG data^83^ and EEG data were re-referenced to the average reference. The reference electrode FCz was added to the signal array.

Next, data were exported from the BrainVision Analyzer and further analyzed in Matlab (Mathworks, Natick, MA) with the FieldTrip^51^ and Brain Connectivity Toolbox,^57^ along with custom-written code. Data were segmented into 2-second epochs with 1-second overlap. A 2-second epoch length was chosen to balance the stationarity of the signals and the number of samples for lower frequencies (down to 4 Hz).^11,73^ All analyses are based on these epochs and the following 4 frequency bands: theta (4-8 Hz), alpha (8-13 Hz), beta (14-30 Hz), and gamma (60-100 Hz). We observed strong nonstationary line noise around 45-55 Hz and therefore excluded the “low gamma” band from frequency band–specific analyses.

### 2.4. Electroencephalography analysis—overview

**Figure 1.**
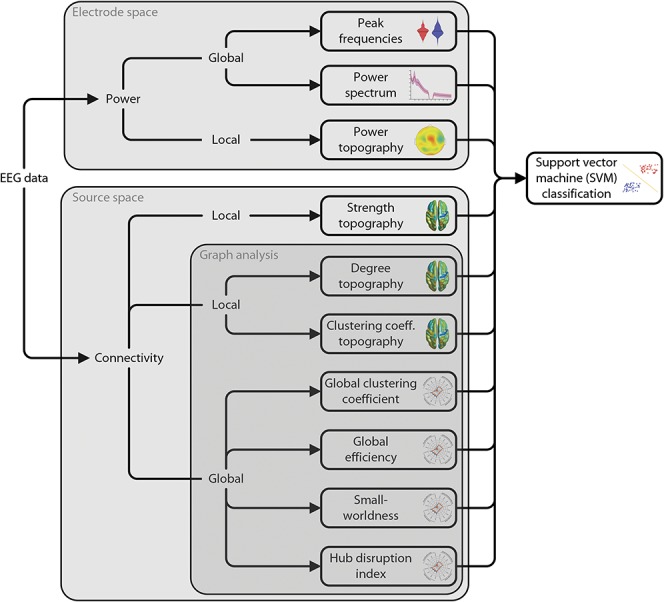
Analysis pipeline. Electroencephalography data were analyzed with regards to power and connectivity, which quantify neural activity and neural communication, respectively. Power analyses were performed in electrode space. Analyses of functional connectivity were performed in source space. Connectivity analyses comprised phase-based (PLV, dwPLI) and amplitude-based (AEC) connectivity measures. Graph–theoretical network analysis was applied to further characterize functional connectivity. All measures were compared between chronic pain patients and healthy controls. In addition, a purely data-driven machine learning approach was adopted, using SVMs. The SVM was trained on all power and connectivity measures to distinguish between chronic pain patients and healthy controls. dwPLI, debiased weighted phase lag index; PLV, phase locking value; SVM, support vector machine.

Figure 1 summarizes the analyses of the EEG data. The analyses included measures of oscillatory brain activity (power) and connectivity in electrode and source space, respectively. Two categories of analyses were performed. First, local, ie, spatially specific, analyses were performed in which a single value is obtained for every electrode, voxel, or brain region. These analyses included comparisons of power topographies on electrode level and comparisons of connectivity and local network measures topographies (degree and clustering coefficient [CC]) on source level. Second, global, ie, spatially holistic, analyses were performed. These analyses include all analyses that average across all electrodes, voxels, or brain regions, ie, the peak frequency, the power spectrum, and all global network measures (see below). In all global analyses, each participant is represented by a single scalar value per measure. All analyses were based on 2-second epochs of resting-state data to balance robustness, frequency resolution, and nonstationarity of the data.

### 2.5. Brain activity (power) analysis

Oscillatory brain activity (power) was analyzed in electrode space. Frequency-specific global power was computed for all epochs using a Fast Fourier Transformation with Slepian multitapers^68^ and a frequency smoothing of 1 Hz and then averaged across epochs and electrodes. Power was first computed for the complete power spectrum, ie, 1 to 100 Hz, with a frequency resolution of 0.5 Hz. To remove line noise, a band-stop filter of 45 to 55 Hz was applied before computing power.

The individual dominant peak frequency was determined on the average of the epochs as the highest local maximum (larger than its 2 neighboring samples) of the amplitude in the frequency range of 6 to 14 Hz.^5^ We also pursued alternative approaches to determine the peak frequency by (1) computing the center of gravity of the power spectrum,^5,36^ (2) computing the dominant peak frequency using longer time windows of 5 seconds, and by (3) computing the peak frequency on each single epoch and then averaging the peaks.^26^

To compare the spatial distribution of local brain activity between patients and healthy controls, power was averaged within each frequency band before comparing frequency-specific power topographies between groups using cluster-based permutation tests.

Relative power was calculated by normalizing every power value (both local and global power) by the respective participant's total power. Total power was calculated by summing all power values across frequencies from 1 to 100 Hz and across all electrodes.

### 2.6. Connectivity analysis

Connectivity analyses were performed in source space. For source analysis, we used linearly constrained minimum variance beamforming^74^ to project the band-pass filtered data for each frequency band and participant from electrode space into source space. This was done with a combination of the FieldTrip toolbox^51^ and custom-written code. Spatial filters for every single person were computed based on the covariance matrices of the band-pass filtered data for each frequency band and a lead field matrix. A 3-dimensional grid with a 1-cm resolution covering the brain was defined, resulting in a total of 2020 voxels in the brain. The lead field was constructed for each voxel using a realistically shaped 3-shell boundary-element volume conduction model based on the template Montreal Neurological Institute (MNI) brain. We used a regularization parameter of 5% of the covariance matrix and chose the dipole orientation of most variance using singular value decomposition. Finally, the preprocessed and band-pass filtered EEG data of each subject were projected through the individual spatial filter to extract the amplitude time series of neuronal activity of each frequency band at each voxel.

Connectivity analyses of EEG data were performed using phase-based and amplitude-based approaches that likely capture different and complementary neural communication processes (see Ref. 20 for a review of these approaches and communication processes). In brief, amplitude-based connectivity is believed to be more closely related to structural connectivity and putatively regulates activation of brain regions.^20^ By contrast, phase-based connectivity seems more detached from structure and more strongly affected by contextual factors.^20^ Here, functional connectivity was investigated using the phase locking value (PLV),^40^ the debiased weighted phase lag index (dwPLI),^78^ and the orthogonalized amplitude envelope correlation (AEC).^32^ The PLV and dwPLI are based on the phase of the signals, whereas the AEC is based on the amplitude. The PLV is well established, highly sensitive, and captures both zero phase lag and nonzero phase lag connectivity. The PLV is sensitive to volume conduction effects because volume conduction can yield spurious synchrony at zero phase lag. Source space analysis can significantly reduce these volume conduction effects on PLV-based connectivity.^29^ Moreover, group contrasts of connectivity further reduce the likelihood of volume conduction effects because these effects are unlikely to differ between groups. The dwPLI captures nonzero phase lag connectivity only. The dwPLI is therefore not susceptible to volume conduction at the cost of reduced sensitivity because real synchrony at zero phase lag is also discarded. This similarly applies to connectivity analyses in electrode space and source space. Because of the explorative character of our study, we mainly report the more sensitive measure, the PLV.

For the connectivity analyses, the connectivity between every pair of voxels was computed, resulting in a 2020 × 2020 connectivity matrix, with a single value representing the strength of connection between 2 voxels over the complete recording time. All 3 connectivity measures are undirected.

### 2.7. Graph–theoretical network analysis

By applying graph–theoretical methods to the data, we reduced the high-dimensional EEG data to a few network measures, characterizing the organization of the whole brain network. Graph theory defines networks as collections of *nodes* and *edges* connecting the *nodes* to each other. We defined the *nodes* as voxels and the *edges* as thresholded functional connectivity between voxels. The connectivity matrix was thresholded to the 10% (5%, 20%) strongest connections and binarized. A binary connectivity matrix was computed to reduce the computational load and to facilitate interpretation^23^ and comparison with previous connectivity analyses.

We used common graph measures that characterize either a single node (local analyses) or the complete network (global analyses).^57^ We investigated 2 local network measures: the *degree* and the *local CC*. The *degree* is the number of a node's edges, ie, the number of connections to other nodes. The *local* CC is the fraction of the node's neighbors that are also neighbors of each other. Thus, both local measures depict how well connected a node is. In particular, the degree indicates how well connected a node is to all other nodes of the network, and the local CC indicates how well connected a node is to neighboring nodes. Four global network measures were included in the analysis: the *global CC* (gCC), *global efficiency* (gEff), *small-worldness* (S), and *hub disruption index* (k_d_).^1^ The *gCC* is the average of the local CC of all nodes indicating the prevalence of clustered connectivity around individual nodes.^57^ In functional brain networks, it is commonly regarded as a measure of functional segregation.^57^ The *global efficiency* is the inverse of the average shortest path length. In functional brain networks, it represents a measure of functional integration.^57^ *Small-worldness* describes the ratio of *CC* and *global efficiency* and compares it with random networks. In functional brain networks, it can quantify the balance of functional segregation and integration^57^ and has been associated with the overall efficiency of communication in a network.^82^ Finally, the *hub disruption index* compares the *degree* of all nodes with those of a control group. Positive values indicate that strongly connected nodes increase and weakly connected nodes decrease their number of connections (“the rich get richer and the poor get poorer”). Conversely, negative values indicate that strongly connected nodes decrease and weakly connected nodes increase their number of connections (“the rich get poorer and the poor get richer”), which means a shift of the network towards a random network with less internal structure.

### 2.8. Correlation analysis

Pearson's *r* was computed between clinical parameters and brain measures, which were found to show significant relationships either in previous studies^10,17,21,28,39,45,58,59,67,75^ or our own. The global peak frequency, mean global power in the 4 frequency bands, hub disruption index and the mean theta and gamma PLV connectivity (averaged across voxels of clusters with significant differences between patients and controls), the PLV global efficiency in the gamma band, and the dwPLI hub disruption index in the gamma band were correlated with the following clinical parameters: current pain intensity, average pain intensity in the past 4 weeks quantified by a visual analogue scale, pain duration, pain disability quantified by the pain disability index,^18^ mental and physical quality of life quantified by the VR-12,^61^ depression quantified by the BDI-II,^6^ and medication as quantified by the medication quantification scale.^30^

### 2.9. Machine learning analysis

The machine learning analysis was conducted using the Statistics and Machine Learning Toolbox in Matlab as well as custom-written scripts. We implemented a support vector machine (SVM)^13^ with a linear kernel, which was trained on all available features. The features were the peak frequency (one feature per participant), global power spectrum (199 features per participant, one feature for each frequency step), local power (4 × 65 features per participant, 65 electrodes), local strength of connectivity (3 × 4 × 2020 features per participant, 2020 voxels in 4 frequency bands and 3 connectivity measures), local degree (3 × 4 × 2020 features per participant, 2020 voxels in 4 frequency bands and 3 connectivity measures), local CC (3 × 4 × 2020 features per participant, 2020 voxels in 4 frequency bands and 3 connectivity measures), and the global graph measures (3 × 4 × 4 features per participant, 4 global graph measures in 4 frequency bands and 3 connectivity measures). This resulted in an SVM containing 73,228 features per participant.

To avoid overfitting, we implemented a so-called sequential forward feature selection. This approach first tests the predictive value of all single features separately. It next takes the most predictive feature and adds a second feature and tests whether the classification improves. This procedure is repeated until no further improvements of the classification can be achieved. In our data, the procedure resulted in a classification that was based on average on 5.5 features rather than more than 70.000 features.

The performance of the SVM was evaluated using a 10-fold cross-validation. First, the data set was randomly split into 10 folds. 9/10 folds were used to train the classifier, with a nested feature selection loop that used another 10-fold cross-validation within the training data to find the most predictive features. Then, the remaining 1/10, which were not included in the training and therefore also not in the feature selection, were classified, resulting in a certain classification accuracy. This procedure was then repeated, cycling through all folds, yielding a mean accuracy over the 10 folds. Because our groups were unbalanced regarding participant numbers, we randomly picked 84 data sets from the cohort of chronic pain patients for the classification procedure, repeating this 1000 times. To conclude whether this result truly exceeds chance level, we repeated the whole procedure with the same data, but the labels of chronic pain patients and healthy controls were randomly shuffled.^12^ Thus, we conducted 2000 analyses: 1000 with 84 randomly picked patients and the 84 healthy controls, and 1000 with the same data but randomly shuffled labels between patients and controls. The 2 resulting distributions of 1000 cross-validation accuracies each were then statistically compared using a nonparametric permutation test.^46^ The sensitivity is defined as the rate of true positives, ie, correctly classified patients, divided by the number of total patient classifications. The specificity is defined as the rate of true negatives, ie, correctly classified healthy controls, divided by the number of total healthy classifications.

Apart from the overall performance of the SVM, we also investigated which features contained the highest predictive value, ie, which features were most consistently picked by the SVMs by looking at the number of times a certain feature was included in the SVM by automatic feature selection.

### 2.10. Statistical analysis

Statistical analysis was performed using FieldTrip^51^ and custom-written Matlab scripts. The significance level for all statistical tests was set to 0.05 two-tailed. The underlying statistical test for all permutation tests was an unpaired T-test. We used cluster-based permutation tests with a cluster threshold of 0.05 to compare patients with healthy controls for all local analyses and the global power spectrum analysis.^46^ We report maximal and minimal T-values (t_max/min) for all cluster-based permutation tests that did not find any clusters. The other global measures were compared using nonparametric permutation tests, permuting between the patient group and healthy control group.^46^ To account for multiple comparisons within a specific measure, we corrected all *P*-values of tests that were performed on all 4 frequency bands using the Holm–Bonferroni method.^33^ To test whether the accuracy of the SVMs was above random chance level, we computed a nonparametric permutation test on the accuracy distribution of the SVM trained on the real data against the accuracy distribution of the SVM trained on the randomly labeled data. Here, a nonparametric permutation test was used to decrease the variety of different statistical tests. Pearson's *r* was calculated to find correlations between brain measures and clinical parameters and tested for statistical significance against the null hypothesis of no correlation. Resulting *P*-values were again corrected for multiple comparisons across the 4 frequency bands using the Holm–Bonferroni method, if applicable.

### 2.11. Data availability

All scripts and data are available on request.

## 3. Results

### 3.1. Global measures of brain activity

**Figure 2.**
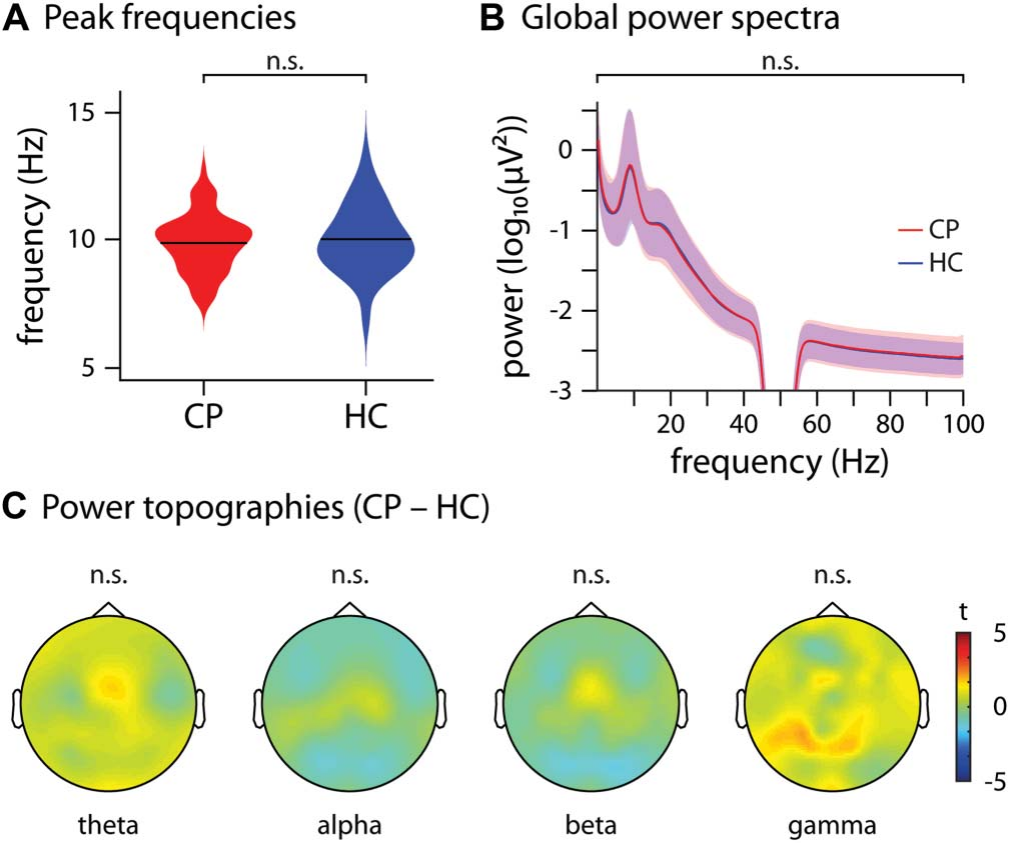
Global and local measures of brain activity. (A) Violin plot of the dominant peak frequencies computed on the average across all electrodes of chronic pain patients (CP, red) and healthy controls (HC, blue). A nonparametric permutation test showed no significant difference (*P* = 0.20) between the 2 groups. (B) Global power spectra of CP (red) and HC (blue), averaged across all electrodes and shown for the frequencies 1 to 100 Hz, with a bandstop filter at 45 to 55 Hz to remove line noise. A cluster-based permutation test clustered across frequencies did not show any significant differences (t_max/min = 1.7/−1.5). (C) Scalp topographies of power differences between CP and HC at theta, alpha, beta, and gamma frequencies, averaged across frequencies in each band. The colormap shows the *t*-values of a cluster-based permutation test. No significant clusters were found in any frequency band (theta t_max/min = 1.5/−0.6, alpha t_max/min = 0.62/−1.2, beta t_max/min = 1.0/−1.4, gamma t_max/min = 2.1/−0.7).

We first investigated whether chronic pain was associated with global changes of oscillatory brain activity. We determined the peak frequency of EEG activity in chronic pain patients and healthy controls by averaging the power spectra across all epochs and electrodes and determining the maximal power in the frequency range of 6 to 14 Hz. Peak frequency was 9.8 ± 1.2 Hz (mean ± SD) in chronic pain patients and 10.0 ± 1.4 Hz in healthy controls and did not differ significantly between groups (nonparametric permutation test, *P* = 0.20, Fig. 2A). Other common approaches to determine the peak frequency, by computing the center of gravity, analyzing longer time windows (5 seconds) for increased spectral resolution, or computing the peak frequency on individual epochs and then averaging, did not show a difference between groups either (nonparametric permutation tests, *P* ≥ 0.14).

Next, we examined whether chronic pain was associated with global changes of oscillatory brain activity at any frequency between 1 and 100 Hz. To this end, we compared the global power spectrum of EEG activity averaged across all electrodes between chronic pain patients and healthy controls. The results did not reveal any significant difference between the 2 groups at any frequency (cluster-based permutation statistics clustered across frequencies, t_max/min = 1.7/−1.5; Fig. 2B). When controlling for intersubject differences in overall power by calculating power relative to the total power across all electrodes and frequencies for each participant, the results did not show a significant difference between patients and controls either (t_max/min = 1.4/−1.7).

Thus, we did not observe global changes of the peak frequency or the power spectrum of oscillatory brain activity in chronic pain patients.

### 3.2. Local measures of brain activity

We further assessed whether chronic pain was associated with local changes of oscillatory brain activity. We therefore calculated topographical maps of brain activity for theta (4-8 Hz), alpha (8-13 Hz), beta (14-30 Hz), and gamma (60-100 Hz) frequency bands. Group comparisons of the topographical maps did not show significant differences between patients and controls in any frequency band (cluster-based permutation statistics clustered across electrodes, t_max/min = 2.0/−1.4, Fig. 2C). When controlling for intersubject differences in overall power by calculating relative power, the results did not show a significant difference between patients and controls either (t_max/min = 2.5/−3.2).

Thus, our findings did not show local changes of oscillatory brain activity in chronic pain patients in any frequency band.

### 3.3. Local measures of functional connectivity

**Figure 3.**
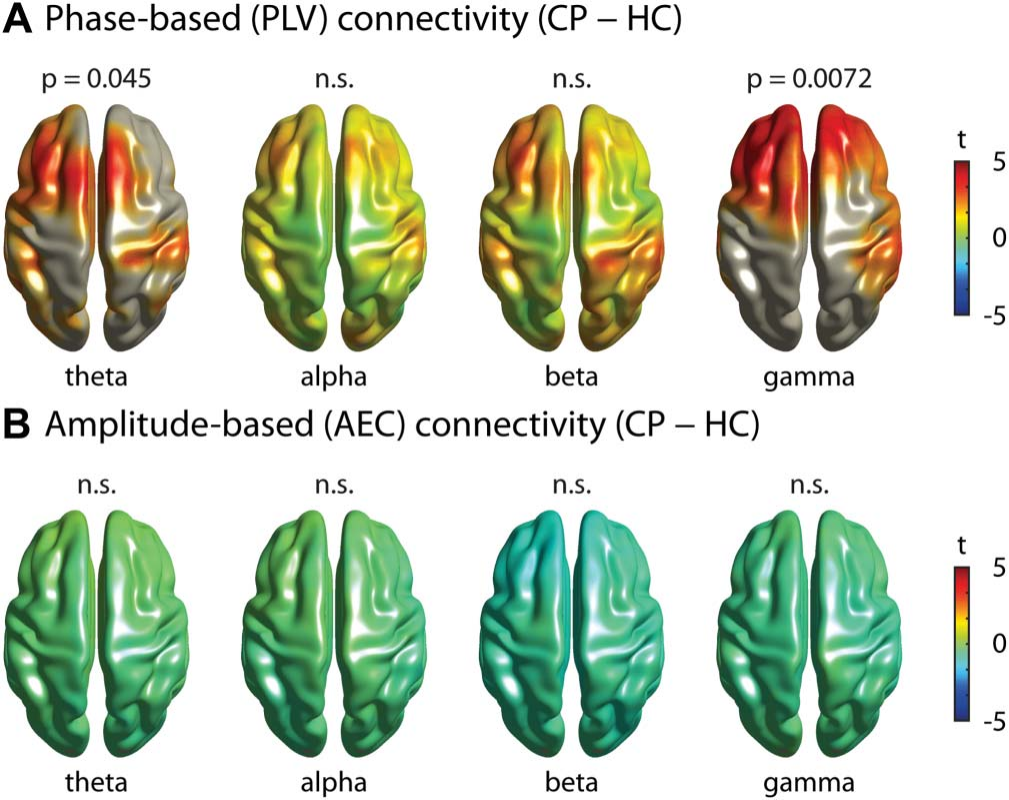
Local measures of functional connectivity. Brain topographies of the comparison of connectivity strength between chronic pain patients (CP) and healthy controls (HC) in the theta, alpha, beta, and gamma band frequencies, averaged across frequencies in each band, are shown. Connectivity strength was calculated as the average connectivity of one voxel to all other voxels of the brain. The colormaps show the *t*-values. Significant results are masked, ie, all voxels but the ones belonging to a significant cluster are grayed out. When no significant clusters are found, nothing is grayed out to show potential trends. (A) Phase-based connectivity (phase locking value, PLV). A significant increase of chronic pain patients' connectivity strength was observed in the theta band (*P* [corrected/uncorrected] = 0.045/0.011, t_max = 3.8, Cohen's *d* = 0.40) and the gamma band (*P* [corrected/uncorrected] = 0.0072/0.0018, t_max = 5.1, Cohen's *d* = 0.59). (B) Amplitude-based connectivity (orthogonalized amplitude envelope correlation, AEC). No significant differences were found in any frequency band (theta t_max/min = 0.4/−0.6, alpha t_max/min = 0.1/−0.7, beta t_max/min = −0.3/−1.2, gamma t_max/min = 0.0/−1.1).

Next, we explored whether chronic pain was associated with changes of functional connectivity, which is a measure of neural communication. To reduce potential confounds by field spread and volume conduction effects,^60^ we performed all connectivity analyses in source space using 2020 voxels with a size of 1 × 1 × 1 cm^3^. We first investigated whether chronic pain was associated with local changes of functional connectivity in any brain region or any frequency band. To this end, we calculated the *connectivity strength* for each voxel and frequency band. *Connectivity strength* was defined as the average connectivity of a specific voxel to all other voxels of the brain, which yields one *connectivity strength* value for each voxel. This allows for visualizing *connectivity strength* in a topographical map and applying the same statistical approaches used for the analysis of local brain activity. Analysis of phase-based connectivity (Fig. 3A) showed that chronic pain patients' *connectivity strength* in the theta band was significantly increased (cluster-based permutation test, *P* [corrected/uncorrected] = 0.045/0.011, Cohen's *d* = 0.40) in comparison with the control group. The strongest contrast was found in the supplementary motor area (MNI = [−10, 10, 70]). Moreover, we also found that patients showed a significantly increased *connectivity strength* in the gamma band (cluster-based permutation test, *P* [corrected/uncorrected] = 0.0072/0.0018, Cohen's *d* = 0.59), which was maximal in the anterior prefrontal cortex (MNI = [−40, 40, 30]). Because in both frequency bands only a single extended cluster of increased connectivity was found, the increase likely reflects frontal connectivity within the clusters rather than connectivity to targets outside of the clusters. No significant clusters were found in the alpha and beta bands (alpha: *P*_min [corrected/uncorrected] = 1/0.61, t_max = 2.8; beta: *P*_min [corrected/uncorrected] = 0.71/0.18, t_max = 3.5). Analysis of amplitude-based connectivity did not show any significant differences in *connectivity strength* between chronic pain patients and healthy controls in any brain region or any frequency band. (Fig. 3B, t_max/min = 0.4/−1.2).

To further assess connectivity patterns of chronic pain patients, we performed graph theory–based network analysis. We first examined the local properties of brain networks in chronic pain patients. A basic property of a node is the number of its connections to other nodes, which is termed the *degree*. Conceptually, the *degree* is closely related to the *connectivity strength* analyzed in the previous step, the essential difference being that the edges are thresholded, whereas the *connectivity strength* considers all connections. We computed the *degree* of every voxel and compared it between patients and controls. No difference in *degree* was found in any frequency band. This applied similarly to phase-based and amplitude-based measures of connectivity (PLV: *P*_min [corrected/uncorrected] = 0.51/0.13, t_max = 4.2; AEC: *P*_min [corrected/uncorrected] = 1/0.56, t_max = 3.0). In addition, we computed the *weighted degree*, ie, a thresholded but not binarized connectivity matrix. Cluster-based permutation tests of the *weighted degree* showed no significant differences between patients and controls in any frequency band either (PLV: *P*_min [corrected/uncorrected] = 0.24/0.061, t_max = 4.3; AEC: *P*_min [corrected/uncorrected] = 1/0.31, t_max = 3.2). This lack of a difference in (weighted) *degree* indicates that the difference in *connectivity strength* is not confined to the strongest connections but instead applies to connections of all strengths.

Another well-established measure that characterizes nodes is the *CC*. This measure assesses the number of connections of neighboring nodes, ie, it measures the local integration of a node served by short-range connectivity. Comparing the *CCs* of all nodes between patients and controls did not show any significant differences at any frequency band, neither for phase-based nor amplitude-based connectivity (PLV: *P*_min [corrected/uncorrected] = 0.12/0.030, t_max = 3.3, AEC: *P*_min [corrected/uncorrected] = 1/0.28, t_max = 3.8).

Taken together, the analysis of local measures of functional connectivity showed increases of phase-based connectivity in frontal and prefrontal cortices at theta and gamma frequencies in chronic pain patients. The increase in the theta band was of small effect size (Cohen's *d* = 0.40), whereas the increase in the gamma band was of medium effect size (Cohen's *d* = 0.59).

### 3.4. Global measures of functional connectivity

**Figure 4.**
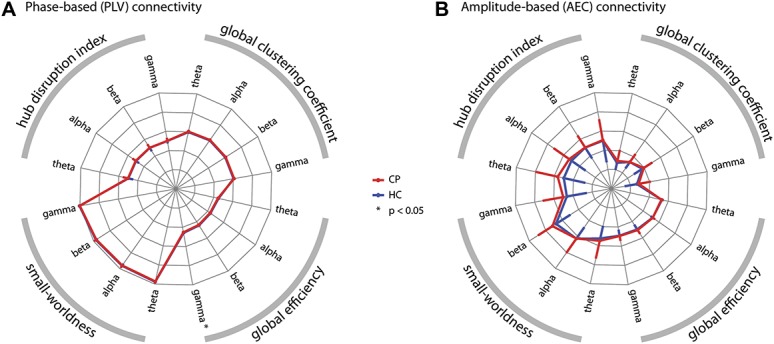
Global graph theoretical measures of functional connectivity. The radar plots show 4 global graph measures in 4 frequency bands based on (A) phase-based and (B) amplitude-based connectivity measures. The clockwise arrangement follows the following pattern: theta, alpha, beta, and gamma repeated for the 4 graph measures: global clustering coefficient, global efficiency, small-worldness, and absolute values of the hub disruption index. The red lines show the chronic pain patients' (CP) values, whereas the blue lines represent the healthy controls' (HC) values. Error bars show the SD. For visualization purposes, the symmetric error bars are only drawn in a single radial direction. Axes run from the center (=0) to the outside (=1). For visualization purposes, the small-worldness and hub disruption index were scaled with a factor of 0.2. (A) Phase-based connectivity (phase locking value, PLV). The global efficiency in the gamma band was significantly decreased in chronic pain patients (nonparametric permutation test, *P* [corrected/uncorrected] = 0.013/0.0032, Cohen's *d* = 0.44). No other measure revealed a significant difference when compared between groups, see Table 3 for details. (B) Amplitude-based connectivity (orthogonalized amplitude envelope correlation, AEC). No significant difference between groups was observed, Table 3 for statistical details.

We next investigated whether chronic pain was associated with changes of global measures of functional connectivity and therefore computed graph measures that characterize different and complementary global properties of brain networks. Figure 4 summarizes the results of the global graph measures. First, we calculated the *gCC*, which is commonly interpreted as a measure of functional segregation in a network. We found no differences in *gCC* between chronic pain patients and healthy controls (Table 3; *P*_min [corrected/uncorrected] = 0.088/0.022). Second, we assessed the *global efficiency,* which provides an account of the ease of long-distance communication and is commonly interpreted as a measure of functional integration in a network. After accounting for multiple comparisons, we found evidence for a decrease of *global efficiency* in patients in the gamma frequency band when investigating phase-based connectivity (Table 3; *P* [corrected/uncorrected] = 0.013/0.0032). The effect size of this difference was small (Cohen's *d* = 0.44). Third, we computed the *small-worldness,* which is associated with communication efficiency within a network. We detected no changes in *small-worldness* between the 2 groups (Table 3; *P*_min [corrected/uncorrected] = 0.26/0.064). Fourth, we analyzed the *hub disruption index*, which has been shown to differ between chronic pain patients and controls in previous functional magnetic resonance imaging studies.^44,45^ It shows potential shifts of connections that manifest on a global scale. Our results did not show a difference of the *hub disruption index* in any frequency band when comparing chronic pain patients with healthy controls (Table 3; *P*_min [corrected/uncorrected] = 1/0.32).

**Table 3:**
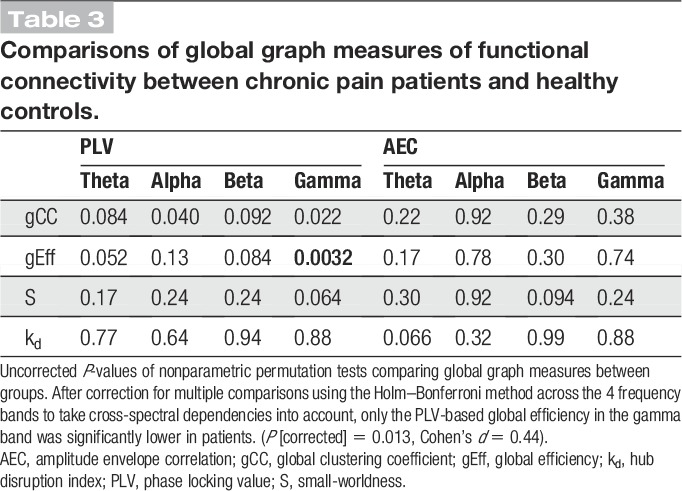
Comparisons of global graph measures of functional connectivity between chronic pain patients and healthy controls.

In summary, global graph measures of functional connectivity showed a decrease of *global efficiency* at gamma frequencies in chronic pain patients. This decrease was of small effect size (Cohen's *d* = 0.44).

### 3.5. Additional functional connectivity analyses

First, as muscle artifacts may cause spurious synchrony, we have analyzed connectivity between EMG signals and EEG signals. Electromyography signals were recorded from the right neck and masseter muscles and band-pass filtered at theta (4-8 Hz) and gamma (60-100 Hz) frequencies. Electroencephalography signals were band-pass filtered source signals from peak voxels of clusters where statistically significant group differences in EEG connectivity were found at theta (MNI coordinates = [−10 10 70]) and gamma (MNI coordinates = [−40 40 30]) frequencies. Statistical comparisons of PLV-based EMG-EEG connectivity did not show any significant differences between chronic pain patients and healthy controls. In particular, nonparametric permutation tests comparing EMG-EEG connectivity band-pass filtered in the theta band (4-8 Hz) between groups yielded *P*-values of 0.3 (neck muscles) and 0.08 (masseter). Group comparisons of EMG-EEG connectivity band-pass filtered in the gamma band (60-100 Hz) yielded *P*-values of 0.6 (neck muscles) and 0.7 (masseter). These findings argue against a contamination of the observed connectivity differences between chronic pain patients and healthy controls by muscle artifacts.

Second, we tested whether changes of functional connectivity in chronic pain patients can be detected using another common phase-based connectivity measure (dwPLI).^66,78^ The dwPLI differs from the PLV by capturing nonzero phase lag connectivity only. The dwPLI is therefore not susceptible to volume conduction, which can yield artificial connectivity effects at the cost of reduced sensitivity because real synchrony at zero phase lag is also discarded. The results of the cluster-based permutation tests did not reveal any local difference in functional connectivity between patients and controls (*P*_min [corrected/uncorrected] = 0.48/0.12, t_min = −3.0). This indicates that zero phase lag connectivity plays an important role in the increased frontal connectivity of patients. Concerning global graph measures (Table 4), the *hub disruption index* was significantly lower in chronic pain patients in the gamma band (*P* [corrected/uncorrected] <0.001/<0.001, Cohen's *d* = 0.63). This difference together with a lack of a difference in the corresponding PLV-based analysis indicates that the difference mainly applies to nonzero phase lag connectivity.

**Table 4:**
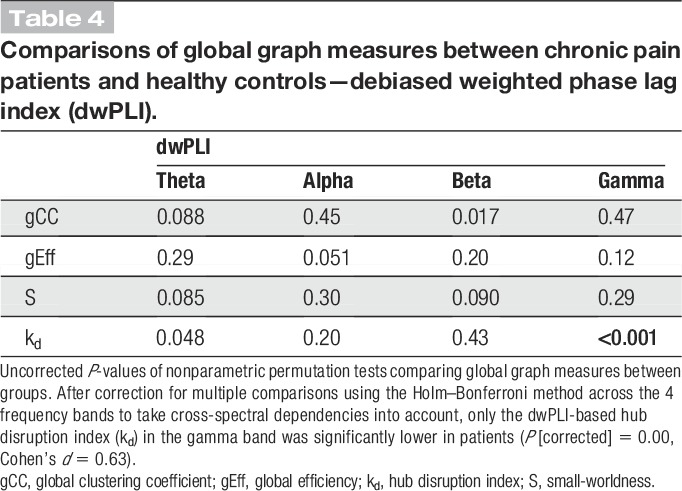
Comparisons of global graph measures between chronic pain patients and healthy controls—debiased weighted phase lag index (dwPLI).

Third, we performed control network analyses by calculating all graph measures with different edge densities using all 3 connectivity measures. This was done to examine the robustness of our results, which were based on a threshold of 10% strongest connections. No significant group differences in local graph measures were found for any of the connectivity measures. Regarding global measures, the *global efficiency* in the gamma band calculated with the PLV (5% edge density, *P* [corrected] = 0.011, Cohen's *d* = 0.44; 20% edge density, *P* [corrected] = 0.0080, Cohen's *d* = 0.44) and the hub disruption index in the gamma band calculated with the dwPLI (5% edge density, *P* [corrected] <0.001, Cohen's *d* = 0.61; 20% edge density, *P* [corrected] <0.001, Cohen's *d* = 0.61) were both significantly lower in chronic pain patients irrespective of edge density and therefore showed a consistent and robust effect.

Finally, we tested whether depression plays a critical role in explaining differences between patients and controls. We therefore aimed to replicate the significant findings after excluding patients with a clinically relevant depression (BDI-II score ≥18, n = 36). This reanalysis did not qualitatively change any of the previously significant results. Frontal connectivity was increased for chronic pain patients without depression in the theta (*P* [corrected] = 0.0080, Cohen's *d* = 0.65) and gamma (*P* [corrected] = 0.0048, Cohen's *d* = 0.68) bands. Similarly, PLV-based global efficiency (*P* [corrected] = 0.014, Cohen's *d* = 0.42) and the dwPLI-based hub disruption index (*P* [corrected] <0.001, *d* = 0.75) were decreased at gamma frequencies in chronic pain patients without depression.

In summary, the PLV *global efficiency* and the dwPLI *hub disruption index* in the gamma band were consistently changed in chronic pain patients even when excluding patients with depression. Both measures were decreased in chronic pain patients, the PLV global efficiency showing a small effect size and the dwPLI hub disruption index showing a medium effect size.

### 3.6. Relationship between brain activity/functional connectivity and clinical parameters

**Figure 5.**
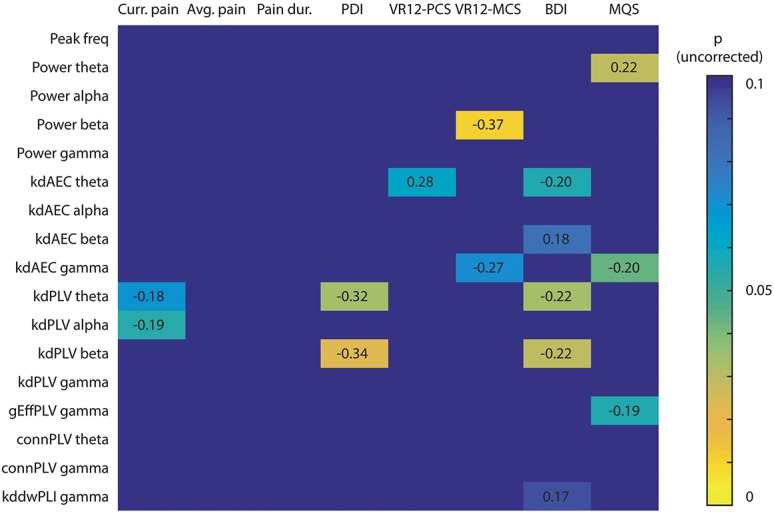
Correlations between clinical/behavioral parameters and brain activity/functional connectivity measures. The cell values show the strength and direction of the correlations (Pearson's *r*) and the color depicts the uncorrected *P* values. Only correlations showing a trend (*P* < 0.1) are shown. No correlation was statistically significant after Holm–Bonferroni correction for multiple comparisons across the 4 frequency bands. AEC, measure is based on the orthogonalized amplitude envelope correlation; Avg. pain, average pain intensity in the past 4 weeks; BDI, Beck Depression Inventory II; conn, connectivity strength; Curr. pain, current pain intensity; dwPLI, measure is based on the debiased weighted phase lag index; gEff, global efficiency; k_d_, hub disruption index; Pain dur., pain duration; peak freq, peak frequency; PDI, pain disability index; PLV, measure is based on the phase locking value; VR12-MCS, Veterans's RAND mental component score; MQS, medication quantification scale; VR12-PCS, Veterans's RAND physical component score.

We further investigated the relationships of brain-based activity and connectivity measures with clinical parameters. To reduce the number of statistical tests, we restrained our analyses to selected measures of brain activity and brain connectivity, which were associated with clinical parameters of chronic pain patients in previous studies.^10,17,21,28,39,45,58,59,67,75^ We thus computed correlations between the global peak frequency, mean global power in the 4 frequency bands, the *hub disruption index*, and the following major clinical parameters: current pain intensity, average pain intensity in the past 4 weeks, pain duration, pain disability, mental and physical quality of life, depression, and medication as quantified by the medication quantification scale. In addition, we correlated the significant clusters in the theta and gamma PLV connectivity, the PLV *global efficiency* in the gamma band, and the dwPLI *hub disruption index* with the same clinical parameters. The results showed no significant correlations (Fig. 5). Thus, we did not observe any relations of measures of brain activity and functional connectivity with clinical parameters including medication. This suggests that increases of frontal connectivity and global network changes in chronic pain patients do not scale with disease characteristics but rather characterize the state of chronic pain per se.

### 3.7. Machine learning approach

**Figure 6.**
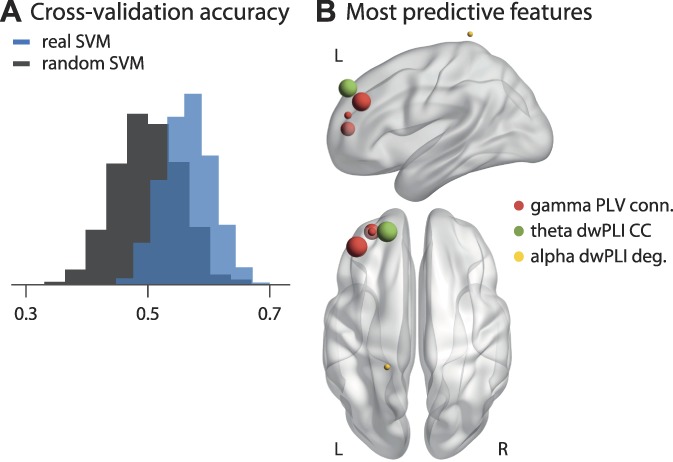
Multivariate machine learning approach to classify chronic pain patients and healthy controls. (A) Distribution of mean accuracies resulting from a 10-fold cross-validation. The blue histogram shows the results trained on the actual data including all features of brain activity and connectivity. The gray histogram shows a support vector machine (SVM) trained on data with randomly permuted labels. The SVM trained on the real data shows an accuracy of 57 ± 4%, significantly higher than the accuracy of the SVM trained on randomly permuted data, 50 ± 5% (*P* < 0.001). (B) The 5 most predictive features, ie, those selected most consistently by the SVMs. Specific measures are color-coded, and the size of the spheres represents how often a specific feature was selected. The most frequently selected features were phase locking value (PLV)-based connectivity of the prefrontal cortex (MNI: −40, 30, 40 and −30, 50, 10) in the gamma band, which were selected in 15% and 12% of SVMs, respectively, and debiased weighted phase lag index (dwPLI) based clustering coefficient of the prefrontal cortex (MNI: −20, 50, 40) in the theta band, which was selected in 15% of SVMs. All other features were selected with a frequency of less than 10%. MNI, Montreal Neurological Institute.

Finally, we performed a multivariate machine learning approach. This approach extends the previous univariate approaches by taking patterns of brain activity and connectivity into account rather than single pieces of information in isolation. Moreover, it complements the previous descriptive group analyses by adding a predictive, single-subject analysis. We used an SVM classifier to test whether patterns of brain activity and/or connectivity can distinguish between chronic pain patients and healthy controls. We trained a linear SVM on all aforementioned measures of brain activity and functional connectivity, using an automated sequential feature selection algorithm. The performance of the SVM was evaluated using a 10-fold cross-validation. The resulting mean accuracy was 57% ± 4% with a sensitivity of 60% ± 5% and a specificity of 57% ± 5%. To test whether this result exceeds chance level, we repeated the whole procedure with the same data but randomly shuffled labels of chronic pain patients and healthy controls. This resulted in a permutation distribution with 50% ± 5% accuracy. A nonparametric permutation test of the 2 accuracy distributions (Fig. 6A) confirmed that the real model was significantly more accurate than random guessing (*P* < 0.001). Finally, we were interested to know which features of brain activity and/or connectivity were most relevant for the classification. The automatic feature selection on average picked 5.5 features for the SVMs. We therefore show the 5 most frequently picked features in Figure 6B. These features were chosen with a rate of 10% to 15% each and all 5 are measures of phase-based connectivity (PLV or dwPLI). Thus, in more than 50% of classifications, phase-based connectivity in frontal brain areas was chosen by the SVMs. The most relevant features were phase-based connectivity measures in frontal brain areas at gamma (MNI: −40, 30, 40 and −30, 50, 10) and theta (MNI: −20, 50, 40) frequencies.

Thus, a multivariate machine learning approach could statistically significantly distinguish between chronic pain patients and healthy controls based on EEG measures of brain activity and connectivity. In particular, frontal phase-based connectivity at theta and gamma frequencies provided important information for the classification.

## 4. Discussion

In this study, we systematically exploited the potential of EEG to determine abnormalities of brain function in chronic pain. Defining such abnormalities promises to advance the understanding of the neural basis of chronic pain. Moreover, they might serve as a brain-based marker and novel treatment target of chronic pain. To this end, we analyzed resting-state EEG recordings of a large cohort of patients suffering from chronic pain and compared them with those of age- and sex-matched healthy controls. The analyses ranged from simple global measures of brain activity to sophisticated connectivity and network analyses in source space. All analyses were data-driven and each analysis was rigorously corrected for multiple comparisons. To the very best of our knowledge, this approach represents the most extensive analysis of EEG data from one of the largest cohorts of chronic pain patients so far. The results show that global measures of brain activity and brain connectivity as measured by EEG did not differ between chronic pain patients and a healthy control group. However, our approach revealed a stronger phase-based connectivity at theta and gamma frequencies in the prefrontal cortex of chronic pain patients. Furthermore, we observed a global reorganization of brain networks in the gamma frequency band. Based on patterns of brain activity and connectivity, a multivariate machine learning approach could classify chronic pain patients and healthy controls with an accuracy of 57%.

Previous resting-state EEG studies investigating alterations in chronic pain patients mainly reported an increase in theta power together with a slowing of the global peak frequency compared with healthy controls.^7,58,76,79^ These findings have been related to the TCD model of chronic pain.^41,42^ In this model, abnormal nociceptive input causes abnormal thalamic bursts at theta frequencies. These theta oscillations are transmitted to the cerebral cortex where they result in disinhibition of neighboring areas, abnormal oscillations at gamma frequencies, and eventually in ongoing pain. This model is highly appealing, but evidence is still sparse. The present completely data-driven approach in a large cohort of chronic pain patients neither shows increased theta power nor a shift of global peak frequency and therefore does not directly support the TCD model of chronic pain.

The univariate comparisons of brain activity/connectivity between groups and the multivariate machine learning approach congruently indicated increased functional connectivity of the prefrontal cortex in chronic pain patients. These findings are in accordance with fMRI^3,31,72^ and EEG^47^ studies as well as with recent reviews and theories,^2,55,62^ which have shown that structural and functional alterations in the prefrontal cortex play an important role in chronic pain. A more precise localization of the connectivity increases in the prefrontal cortex is beyond the spatial resolution of EEG. Hence, it remains unclear how the present observations relate to the multitude of functions represented in the prefrontal cortex, which include motor, cognitive control, emotional, evaluative, and modulatory functions.^16,37^ However, a role of the prefrontal cortex in chronic pain points to an important function of emotional-evaluative, motivational, and decision-making circuits rather than sensory circuits in chronic pain.^2,55^

Our findings revealed that chronic pain is associated with frontal connectivity increases and a global disturbance of brain network organization at gamma frequencies. Mechanistically, gamma oscillations have been related to the activity of inhibitory parvalbumin-positive GABAergic interneurons.^9^ In an animal model of chronic pain, these interneurons have been implicated in the modulation of pyramidal cell firing in the prefrontal cortex and pain behavior.^85^ This link between GABAergic inhibition, gamma oscillations, prefrontal cortex activity, and pain behavior is in accordance with the present observations. Functionally, gamma oscillations likely represent a basic feature of neuronal signaling and communication,^19,25,81^ which seems to be particularly related to the local processing and feedforward communication of currently important stimuli.^19,25,53^ These concepts would be in line with an association of chronic pain with prefrontal gamma oscillations possibly signaling the emotional, motivational, and evaluative aspects of pain.

In addition, decreases of global efficiency and hub disruption index in the gamma band indicate that chronic pain is not only associated with local changes of functional connectivity but also a global reorganization of brain networks. This is in accordance with recent fMRI studies that have shown global changes of functional connectivity at infraslow frequencies below 0.1 Hz.^44,45^ The present findings complement these studies by showing that global changes of functional connectivity also occur in the gamma band at much higher frequencies between 60 and 100 Hz.

The present machine learning approach shows that applying an SVM classifier to resting-state EEG data allows to distinguish between chronic pain patients and healthy controls with 57% accuracy. A recent study that pursued a closely related EEG approach showed accuracies of >90% for the classification of chronic pain patients vs healthy controls,^76^ which were not achieved in this study. The reasons for this disparity remain unclear because the available information on the previous approach does not allow for precise replication. Although the accuracy of the present study is far from being sufficient for practical purposes, this result has important implications. First, it indicates that prefrontal synchrony at theta and gamma frequencies might play a role in the pathophysiology of chronic pain. Second, the present approach might represent a step further towards a much sought-after brain-based marker of pain.^15,71^ fMRI recordings have already shown that, in principle, it is possible to establish such a marker.^44,80^ The present approach complements these fMRI approaches by using EEG recordings. Third, abnormal patterns of EEG activity in chronic pain might represent potential targets for novel therapeutic strategies such as non-invasive brain stimulation techniques^54^ and/or neurofeedback approaches.^63^ In particular, the emerging transcranial alternating current stimulation technique^54^ allows for the frequency-specific modulation of neuronal oscillations and synchrony and might, thus, represent a promising approach to modulate pain.

Several limitations of this study need to be pointed out. First, abnormal oscillations and synchrony are observed in many neurological diseases including depression^69,70^ and the specificity of the present results for chronic pain remains unclear. However, changes of prefrontal theta and gamma synchrony were similarly found when patients with depression were excluded. Moreover, the causal relationship between the observed changes of brain function and chronic pain remains unclear. However, a potential lack of specificity and/or causality does not necessarily limit the clinical usefulness and validity of a brain-based marker of chronic pain.^15^ Second, field spread and/or muscle artifacts can cause spurious synchrony of EEG signals. A rigorous artifact correction procedure and analysis in source space are best practice to reduce these effects. Moreover, the present analyses are based on comparisons between groups, and systematic differences of volume conduction between groups are unlikely. Furthermore, a lack of group differences in amplitude of oscillatory of brain activity argues against volume conduction effects on the observed group differences in connectivity. In addition, a lack of connectivity between EMG and EEG signals argues against a contamination of the observed differences in EEG connectivity by EMG signals. Nevertheless, muscle artifacts and volume conduction remain an inherent and delicate confound of EEG signals. Third, drug effects cannot be ultimately ruled out. We excluded patients taking benzodiazepines, which have known effects on EEG signals.^4^ However, in our representative cohort of chronic pain patients, most patients took nonopioid analgesics, opioids, and/or antidepressants. To control for drug effects, we quantified medication and found no significant correlations between medication and the observed EEG effects. Therefore, it is unlikely but not impossible that our effects are solely driven by drug effects. Fourth, for each measure of brain activity and connectivity, we have corrected for multiple comparisons across frequency and/or space. However, we have not corrected for multiple comparisons across the different measures of brain activity and brain connectivity. This has been done to decrease the risk of false-negative findings. However, it increases the risk of false-positive findings so that the current findings need replication and reproduction.

In conclusion, our data-driven, systematic, and extensive analysis of EEG data from a large cohort of chronic pain patients shows that local and global measures of brain activity did not differ between chronic pain patients and a healthy control group. These negative findings might help to clarify inconsistencies in previous studies and guide future research. Moreover, our study reveals increased prefrontal synchrony together with global network reorganization at gamma frequencies in chronic pain, which allows for differentiating chronic pain patients from healthy controls. These findings advance the understanding of the brain mechanisms of chronic pain. Beyond, the present observations might represent a step further towards a safe, low-cost, broadly available, and potentially mobile brain-based marker of pain. However, substantial challenges concerning the possibility of false-positive findings and the accuracy, specificity, and validity of such a marker remain to be overcome. Finally, the findings might open new therapeutic perspectives by revealing a potential target for novel noninvasive brain stimulation and neurofeedback strategies.

## Disclosures

The authors have no conflicts of interest to declare.

## References

[1] Achard S Delon-Martin C Vértes PE Renard F Schenck M Schneider F Heinrich C Kremer S Bullmore ET Hubs of brain functional networks are radically reorganized in comatose patients. Proc Natl Acad Sci USA 2012;109:20608–13. 23185007 10.1073/pnas.1208933109 PMC3528500

[2] Baliki MN Apkarian AV Nociception, pain, negative moods, and behavior selection. Neuron 2015;87:474–91. 26247858 10.1016/j.neuron.2015.06.005 PMC4529956

[3] Baliki MN Petre B Torbey S Herrmann KM Huang L Schnitzer TJ Fields HL Apkarian AV Corticostriatal functional connectivity predicts transition to chronic back pain. Nat Neurosci 2012;15:1117–9. 22751038 10.1038/nn.3153 PMC3411898

[4] Bauer G Bauer R EEG, drug effects, and central nervous system poisoning. In: Schomer DL da Silva FHL, editors. Niedermeyer's electroencephalography: basic principles, clinical applications, and related fields. Philadelphia: Oxford University Press, 2011 pp. 901–22.

[5] Bazanova OM Vernon D Interpreting EEG alpha activity. Neurosci Biobehav Rev 2014;44:94–110. 23701947 10.1016/j.neubiorev.2013.05.007

[6] Beck AT Steer RA Brown G Manual for the beck depression inventory-II. San Antonio: Psychological Corporation, 1996.

[7] Boord P Siddall PJ Tran Y Herbert D Middleton J Craig A Electroencephalographic slowing and reduced reactivity in neuropathic pain following spinal cord injury. Spinal Cord 2008;46:118–23. 17502876 10.1038/sj.sc.3102077

[8] Breivik H Collett B Ventafridda V Cohen R Gallacher D Survey of chronic pain in Europe: prevalence, impact on daily life, and treatment. Eur J Pain 2006;10:287–333. 16095934 10.1016/j.ejpain.2005.06.009

[9] Buzsáki G Wang XJ Mechanisms of gamma oscillations. Annu Rev Neurosci 2012;35:203–25. 22443509 10.1146/annurev-neuro-062111-150444 PMC4049541

[10] Choe MK Lim M Kim JS Lee DS Chung CK Disrupted resting state network of fibromyalgia in theta frequency. Sci Rep 2018;8:2064. 29391478 10.1038/s41598-017-18999-z PMC5794911

[11] Chu CJ Kramer MA Pathmanathan J Bianchi MT Westover MB Wizon L Cash SS Emergence of stable functional networks in long-term human electroencephalography. J Neurosci 2012;32:2703–13. 22357854 10.1523/JNEUROSCI.5669-11.2012 PMC3361717

[12] Combrisson E Jerbi K Exceeding chance level by chance: the caveat of theoretical chance levels in brain signal classification and statistical assessment of decoding accuracy. J Neurosci Methods 2015;250:126–36. 25596422 10.1016/j.jneumeth.2015.01.010

[13] Cortes C Vapnik V Support-vector networks. Machine learning 1995;20:273–97.

[14] Davis JF Manual of surface electromyography. WADC Technical Report (59-184). Montreal, 1959.

[15] Davis KD Flor H Greely HT Iannetti GD Mackey S Ploner M Pustilnik A Tracey I Treede RD Wager TD Brain imaging tests for chronic pain: medical, legal and ethical issues and recommendations. Nat Rev Neurol 2017;13:624–38. 28884750 10.1038/nrneurol.2017.122

[16] de la Vega A Chang LJ Banich MT Wager TD Yarkoni T Large-scale meta-analysis of human medial frontal cortex reveals tripartite functional organization. J Neurosci 2016;36:6553–62. 27307242 10.1523/JNEUROSCI.4402-15.2016 PMC5015787

[17] de Vries M Wilder-Smith OH Jongsma ML van den Broeke EN Arns M van Goor H van Rijn CM Altered resting state EEG in chronic pancreatitis patients: toward a marker for chronic pain. J Pain Res 2013;6:815–24. 24379694 10.2147/JPR.S50919 PMC3843642

[18] Dillmann U Nilges P Saile H Gerbershagen HU Assessing disability in chronic pain patients [in German]. Schmerz 1994;8:100–10. 18415443 10.1007/BF02530415

[19] Donner TH Siegel M A framework for local cortical oscillation patterns. Trends Cogn Sci 2011;15:191–9. 21481630 10.1016/j.tics.2011.03.007

[20] Engel AK Gerloff C Hilgetag CC Nolte G Intrinsic coupling modes: multiscale interactions in ongoing brain activity. Neuron 2013;80:867–86. 24267648 10.1016/j.neuron.2013.09.038

[21] Fallon N Chiu Y Nurmikko T Stancak A Altered theta oscillations in resting EEG of fibromyalgia syndrome patients. Eur J Pain 2018;22:49–57. 28758313 10.1002/ejp.1076 PMC5763419

[22] Faul F Erdfelder E Lang AG Buchner A G. *Power 3: a flexible statistical power analysis program for the social, behavioral, and biomedical sciences. Behav Res Methods 2007;39:175–91. 17695343 10.3758/bf03193146

[23] Fornito A Zalesky A Bullmore ET Fundamentals of brain network analysis. San Diego: Academic Press, 2016.

[24] Freynhagen R Baron R Gockel U Tölle TR painDETECT: a new screening questionnaire to identify neuropathic components in patients with back pain. Curr Med Res Opin 2006;22:1911–20. 17022849 10.1185/030079906X132488

[25] Fries P Rhythms for cognition: communication through coherence. Neuron 2015;88:220–35. 26447583 10.1016/j.neuron.2015.09.034 PMC4605134

[26] Furman AJ Meeker TJ Rietschel JC Yoo S Muthulingam J Prokhorenko M Keaser ML Goodman RN Mazaheri A Seminowicz DA Cerebral peak alpha frequency predicts individual differences in pain sensitivity. Neuroimage 2017;167:203–10. 29175204 10.1016/j.neuroimage.2017.11.042

[27] Global Burden of Disease Study C. Global, regional, and national incidence, prevalence, and years lived with disability for 328 diseases and injuries for 195 countries, 1990-2016: a systematic analysis for the Global Burden of Disease Study 2016. Lancet 2017;390:1211–59. 28919117 10.1016/S0140-6736(17)32154-2 PMC5605509

[28] Gonzalez-Roldan AM Cifre I Sitges C Montoya P Altered dynamic of EEG oscillations in fibromyalgia patients at rest. Pain Med 2016;17:1058–68. 26921889 10.1093/pm/pnw023

[29] Gross J Baillet S Barnes GR Henson RN Hillebrand A Jensen O Jerbi K Litvak V Maess B Oostenveld R Parkkonen L Taylor JR van Wassenhove V Wibral M Schoffelen JM Good practice for conducting and reporting MEG research. Neuroimage 2013;65:349–63. 23046981 10.1016/j.neuroimage.2012.10.001 PMC3925794

[30] Harden RN Weinland SR Remble TA Houle TT Colio S Steedman S Kee WG Medication Quantification Scale Version III: update in medication classes and revised detriment weights by survey of American Pain Society Physicians. J Pain 2005;6:364–71. 15943958 10.1016/j.jpain.2005.01.350

[31] Hashmi JA Baliki MN Huang L Baria AT Torbey S Hermann KM Schnitzer TJ Apkarian AV Shape shifting pain: chronification of back pain shifts brain representation from nociceptive to emotional circuits. Brain 2013;136:2751–68. 23983029 10.1093/brain/awt211 PMC3754458

[32] Hipp JF Hawellek DJ Corbetta M Siegel M Engel AK Large-scale cortical correlation structure of spontaneous oscillatory activity. Nat Neurosci 2012;15:884–90. 22561454 10.1038/nn.3101 PMC3861400

[33] Holm S A simple sequentially rejective multiple test procedure. Scand J Statist 1979;6:65–70.

[34] Jung TP Makeig S Humphries C Lee TW McKeown MJ Iragui V Sejnowski TJ Removing electroencephalographic artifacts by blind source separation. Psychophysiology 2000;37:163–78. 10731767

[35] Kennedy J Roll JM Schraudner T Murphy S McPherson S Prevalence of persistent pain in the U.S. adult population: new data from the 2010 national health interview survey. J Pain 2014;15:979–84. 25267013 10.1016/j.jpain.2014.05.009

[36] Klimesch W Schimke H Pfurtscheller G Alpha frequency, cognitive load and memory performance. Brain Topogr 1993;5:241–51. 8507550 10.1007/BF01128991

[37] Kragel PA Kano M Van Oudenhove L Ly HG Dupont P Rubio A Delon-Martin C Bonaz BL Manuck SB Gianaros PJ Ceko M Reynolds Losin EA Woo CW Nichols TE Wager TD Generalizable representations of pain, cognitive control, and negative emotion in medial frontal cortex. Nat Neurosci 2018;21:283–9. 29292378 10.1038/s41593-017-0051-7 PMC5801068

[38] Kuner R Flor H Structural plasticity and reorganisation in chronic pain. Nat Rev Neurosci 2017;18:113. 28704354 10.1038/nrn.2017.5

[39] Kuo PC Chen YT Chen YS Chen LF Decoding the perception of endogenous pain from resting-state MEG. Neuroimage 2017;144:1–11. 27746387 10.1016/j.neuroimage.2016.09.040

[40] Lachaux JP Rodriguez E Martinerie J Varela FJ Measuring phase synchrony in brain signals. Hum Brain Mapp 1999;8:194–208. 10619414 10.1002/(SICI)1097-0193(1999)8:4<194::AID-HBM4>3.0.CO;2-C PMC6873296

[41] Llinás R Urbano FJ Leznik E Ramírez RR van Marle HJ Rhythmic and dysrhythmic thalamocortical dynamics: GABA systems and the edge effect. Trends Neurosci 2005;28:325–33. 15927689 10.1016/j.tins.2005.04.006

[42] Llinás RR Ribary U Jeanmonod D Kronberg E Mitra PP Thalamocortical dysrhythmia: a neurological and neuropsychiatric syndrome characterized by magnetoencephalography. Proc Natl Acad Sci USA 1999;96:15222–7. 10611366 10.1073/pnas.96.26.15222 PMC24801

[43] Makin S Imaging: show me where it hurts. Nature 2016;535:S8–9. 27410532 10.1038/535S8a

[44] Mano H Kotecha G Leibnitz K Matsubara T Sprenger C Nakae A Shenker N Shibata M Voon V Yoshida W Lee M Yanagida T Kawato M Rosa MJ Seymour B Classification and characterisation of brain network changes in chronic back pain: a multicenter study. Wellcome Open Res 2018;3:19. 29774244 10.12688/wellcomeopenres.14069.1 PMC5930551

[45] Mansour A Baria AT Tetreault P Vachon-Presseau E Chang PC Huang L Apkarian AV Baliki MN Global disruption of degree rank order: a hallmark of chronic pain. Sci Rep 2016;6:34853. 27725689 10.1038/srep34853 PMC5057075

[46] Maris E Oostenveld R Nonparametric statistical testing of EEG- and MEG-data. J Neurosci Methods 2007;164:177–90. 17517438 10.1016/j.jneumeth.2007.03.024

[47] May ES Nickel MM Ta Dinh S Tiemann L Heitmann H Voth I Tölle TR Gross J Ploner M Prefrontal gamma oscillations reflect ongoing pain intensity in chronic back pain patients. Hum Brain Mapp 2019;40:293–305. 30260531 10.1002/hbm.24373 PMC6585682

[48] Melzack R The short-form McGill pain questionnaire. PAIN 1987;30:191–7. 3670870 10.1016/0304-3959(87)91074-8

[49] Moriarty O McGuire BE Finn DP The effect of pain on cognitive function: a review of clinical and preclinical research. Prog Neurobiol 2011;93:385–404. 21216272 10.1016/j.pneurobio.2011.01.002

[50] Mouraux A Iannetti GD The search for pain biomarkers in the human brain. Brain 2018;141:3290–307. 30462175 10.1093/brain/awy281 PMC6262221

[51] Oostenveld R Fries P Maris E Schoffelen JM FieldTrip: open source software for advanced analysis of MEG, EEG, and invasive electrophysiological data. Comput Intell Neurosci 2011;2011:156869. 21253357 10.1155/2011/156869 PMC3021840

[52] Pinheiro ES de Queirós FC Montoya P Santos CL do Nascimento MA Ito CH Silva M Nunes Santos DB Benevides S Miranda JG Sá KN Baptista AF Electroencephalographic patterns in chronic pain: a systematic review of the literature. PLoS One 2016;11:e0149085. 26914356 10.1371/journal.pone.0149085 PMC4767709

[53] Ploner M Sorg C Gross J Brain rhythms of pain. Trends Cogn Sci 2017;21:100–10. 28025007 10.1016/j.tics.2016.12.001 PMC5374269

[54] Polanía R Nitsche MA Ruff CC Studying and modifying brain function with non-invasive brain stimulation. Nat Neurosci 2018;21:174–87. 29311747 10.1038/s41593-017-0054-4

[55] Rauschecker JP May ES Maudoux A Ploner M Frontostriatal gating of tinnitus and chronic pain. Trends Cogn Sci 2015;19:567–78. 26412095 10.1016/j.tics.2015.08.002 PMC4587397

[56] Reardon S Neuroscience in court: the painful truth. Nature 2015;518:474–6. 25719648 10.1038/518474a

[57] Rubinov M Sporns O Complex network measures of brain connectivity: uses and interpretations. Neuroimage 2010;52:1059–69. 19819337 10.1016/j.neuroimage.2009.10.003

[58] Sarnthein J Stern J Aufenberg C Rousson V Jeanmonod D Increased EEG power and slowed dominant frequency in patients with neurogenic pain. Brain 2006;129:55–64. 16183660 10.1093/brain/awh631

[59] Schmidt S Naranjo JR Brenneisen C Gundlach J Schultz C Kaube H Hinterberger T Jeanmonod D Pain ratings, psychological functioning and quantitative EEG in a controlled study of chronic back pain patients. PLoS One 2012;7:e31138. 22431961 10.1371/journal.pone.0031138 PMC3303776

[60] Schoffelen JM Gross J Source connectivity analysis with MEG and EEG. Hum Brain Mapp 2009;30:1857–65. 19235884 10.1002/hbm.20745 PMC6870611

[61] Selim AJ Rogers W Fleishman JA Qian SX Fincke BG Rothendler JA Kazis LE Updated U.S. Population standard for the Veterans RAND 12-item health survey (VR-12). Qual Life Res 2009;18:43–52. 19051059 10.1007/s11136-008-9418-2

[62] Seminowicz DA Moayedi M The dorsolateral prefrontal cortex in acute and chronic pain. J Pain 2017;18:1027–35. 28400293 10.1016/j.jpain.2017.03.008 PMC5581265

[63] Sitaram R Ros T Stoeckel L Haller S Scharnowski F Lewis-Peacock J Weiskopf N Blefari ML Rana M Oblak E Birbaumer N Sulzer J Closed-loop brain training: the science of neurofeedback. Nat Rev Neurosci 2017;18:86–100. 28003656 10.1038/nrn.2016.164

[64] Smith SM Dworkin RH Turk DC Baron R Polydefkis M Tracey I Borsook D Edwards RR Harris RE Wager TD Arendt-Nielsen L Burke LB Carr DB Chappell A Farrar JT Freeman R Gilron I Goli V Haeussler J Jensen T Katz NP Kent J Kopecky EA Lee DA Maixner W Markman JD McArthur JC McDermott MP Parvathenani L Raja SN Rappaport BA Rice ASC Rowbotham MC Tobias JK Wasan AD Witter J The potential role of sensory testing, skin biopsy, and functional brain imaging as biomarkers in chronic pain clinical trials: IMMPACT considerations. J Pain 2017;18:757–77. 28254585 10.1016/j.jpain.2017.02.429 PMC5484729

[65] Spielberger CD Gorsuch RL Lushene R Vagg PR Jacobs GA Manual for the state-trait anxiety inventory. Palo Alto: Consulting Psychologists Press, 1983.

[66] Stam CJ Nolte G Daffertshofer A Phase lag index: assessment of functional connectivity from multi channel EEG and MEG with diminished bias from common sources. Hum Brain Mapp 2007;28:1178–93. 17266107 10.1002/hbm.20346 PMC6871367

[67] Stern J Jeanmonod D Sarnthein J Persistent EEG overactivation in the cortical pain matrix of neurogenic pain patients. Neuroimage 2006;31:721–31. 16527493 10.1016/j.neuroimage.2005.12.042

[68] Thomson DJ Spectrum estimation and harmonic analysis. Proc IEEE 1982;70:1055–96.

[69] Uhlhaas PJ Singer W Neural synchrony in brain disorders: relevance for cognitive dysfunctions and pathophysiology. Neuron 2006;52:155–68. 17015233 10.1016/j.neuron.2006.09.020

[70] Uhlhaas PJ Singer W Neuronal dynamics and neuropsychiatric disorders: toward a translational paradigm for dysfunctional large-scale networks. Neuron 2012;75:963–80. 22998866 10.1016/j.neuron.2012.09.004

[71] Upadhyay J Geber C Hargreaves R Birklein F Borsook D A critical evaluation of validity and utility of translational imaging in pain and analgesia: utilizing functional imaging to enhance the process. Neurosci Biobehav Rev 2018;84:407–23. 28807753 10.1016/j.neubiorev.2017.08.004 PMC5729102

[72] Vachon-Presseau E Tetreault P Petre B Huang L Berger SE Torbey S Baria AT Mansour AR Hashmi JA Griffith JW Comasco E Schnitzer TJ Baliki MN Apkarian AV Corticolimbic anatomical characteristics predetermine risk for chronic pain. Brain 2016;139:1958–70. 27190016 10.1093/brain/aww100 PMC4939699

[73] van Diessen E Numan T van Dellen E van der Kooi AW Boersma M Hofman D van Lutterveld R van Dijk BW van Straaten EC Hillebrand A Stam CJ Opportunities and methodological challenges in EEG and MEG resting state functional brain network research. Clin Neurophysiol 2015;126:1468–81. 25511636 10.1016/j.clinph.2014.11.018

[74] Van Veen BD van Drongelen W Yuchtman M Suzuki A Localization of brain electrical activity via linearly constrained minimum variance spatial filtering. IEEE Trans Biomed Eng 1997;44:867–80. 9282479 10.1109/10.623056

[75] Vanneste S Ost J Van Havenbergh T De Ridder D Resting state electrical brain activity and connectivity in fibromyalgia. PLoS One 2017;12:e0178516. 28650974 10.1371/journal.pone.0178516 PMC5484465

[76] Vanneste S Song JJ De Ridder D Thalamocortical dysrhythmia detected by machine learning. Nat Commun 2018;9:1103. 29549239 10.1038/s41467-018-02820-0 PMC5856824

[77] Velly AM Mohit S Epidemiology of pain and relation to psychiatric disorders. Prog Neuropsychopharmacol Biol Psychiatry 2018;87(pt B):159–167. 28522289 10.1016/j.pnpbp.2017.05.012

[78] Vinck M Oostenveld R van Wingerden M Battaglia F Pennartz CM An improved index of phase-synchronization for electrophysiological data in the presence of volume-conduction, noise and sample-size bias. Neuroimage 2011;55:1548–65. 21276857 10.1016/j.neuroimage.2011.01.055

[79] Vuckovic A Hasan MA Fraser M Conway BA Nasseroleslami B Allan DB Dynamic oscillatory signatures of central neuropathic pain in spinal cord injury. J Pain 2014;15:645–55. 24589821 10.1016/j.jpain.2014.02.005 PMC4058526

[80] Wager TD Atlas LY Lindquist MA Roy M Woo CW Kross E An fMRI-based neurologic signature of physical pain. N Engl J Med 2013;368:1388–97. 23574118 10.1056/NEJMoa1204471 PMC3691100

[81] Wang XJ Neurophysiological and computational principles of cortical rhythms in cognition. Physiol Rev 2010;90:1195–268. 20664082 10.1152/physrev.00035.2008 PMC2923921

[82] Watts DJ Strogatz SH Collective dynamics of “small-world” networks. Nature 1998;393:440–2. 9623998 10.1038/30918

[83] Winkler I Debener S Müller KR Tangermann M On the influence of high-pass filtering on ICA-based artifact reduction in EEG-ERP. Conf Proc IEEE Eng Med Biol Soc 2015;2015:4101–5. 26737196 10.1109/EMBC.2015.7319296

[84] Woo CW Chang LJ Lindquist MA Wager TD Building better biomarkers: brain models in translational neuroimaging. Nat Neurosci 2017;20:365–77. 28230847 10.1038/nn.4478 PMC5988350

[85] Zhang Z Gadotti VM Chen L Souza IA Stemkowski PL Zamponi GW Role of prelimbic GABAergic circuits in sensory and emotional aspects of neuropathic pain. Cell Rep 2015;12:752–9. 26212331 10.1016/j.celrep.2015.07.001

